# A practical guide and Galaxy workflow to avoid inter-plasmidic repeat collapse and false gene loss in Unicycler’s hybrid assemblies

**DOI:** 10.1099/mgen.0.001173

**Published:** 2024-01-10

**Authors:** Lea Schäfer, Johannes A. Jehle, Regina G. Kleespies, Jörg T. Wennmann

**Affiliations:** ^1^​ Julius Kühn Institute (JKI) – Federal Research Centre for Cultivated Plants, Institute for Biological Control, Schwabenheimer Str. 101, 69221 Dossenheim, Germany

**Keywords:** gene duplication, hybrid assembly, Nanopore, plasmids, repeat, whole-genome sequencing

## Abstract

Generating complete, high-quality genome assemblies is key for any downstream analysis, such as comparative genomics. For bacterial genome assembly, various algorithms and fully automated pipelines exist, which are free-of-charge and easily accessible. However, these assembly tools often cannot unambiguously resolve a bacterial genome, for example due to the presence of sequence repeat structures on the chromosome or on plasmids. Then, a more sophisticated approach and/or manual curation is needed. Such modifications can be challenging, especially for non-bioinformaticians, because they are generally not considered as a straightforward process. In this study, we propose a standardized approach for manual genome completion focusing on the popular hybrid assembly pipeline Unicycler. The provided Galaxy workflow addresses two weaknesses in Unicycler’s hybrid assemblies: (i) collapse of inter-plasmidic repeats and (ii) false loss of single-copy sequences. To demonstrate and validate how to detect and resolve these assembly errors, we use two genomes from the *Bacillus cereus* group. By applying the proposed pipeline following an automated assembly, the genome sequence quality can be significantly improved.

## Abbreviations

Btt, Bacillus thuringiensis subsp. tenebrionis; Bw, Bacillus wiedmannii; gDNA, genomic DNA; ONT, Oxford Nanopore Technology; ten, tenebrionis.

## Impact Statement

Studies and scientists, especially those dealing with sequencing and assembly of complex bacterial genomes for the first time, are often faced with the challenge of incompletely resolved genomes. Not only does the combination of short- (Illumina) and long-read (Nanopore) sequencing data using the assembly tool Unicycler offer a solution, but in particular the understanding of (i) inter-plasmidic repeat collapse and (ii) false loss of single-copy sequences is essential. This study aimed to understand these terms, how they occur in assembly and how to solve them using a Galaxy-based workflow. The provided workflow emphasizes being beginner-friendly, which does not require professional expertise in bioinformatics and command line on Linux to run programs.

## Data Summary

All sequence data generated in this study (Illumina and ONT raw read data, genome assemblies) have been deposited under NCBI BioProject accessions PRJNA886432 and PRJNA1010135, and BioSample accessions SAMN37105267 (*Bacillus thuringiensis* subsp. *tenebrionis* strain ten BI 256-82/CM3) and SAMN37179813 (*Bacillus wiedmannii* strain EPS29). Final genome assemblies, including the chromosome and all plasmids, can be found under accession numbers CP133375–CP133380 (ten BI256-82/CM39) and CP133557-CP133559 (*Bacillus wiedmannii* strain EPS29). All underlying code of the Galaxy workflow is available at https://github.com/wennj/plasmid-collapse-gene-loss-unicycler-galaxy. The authors confirm all supporting data, code and protocols have been provided within the article or through supplementary data files.

## Introduction

The number of fully sequenced bacterial genomes is constantly increasing, as both sequencing technologies and genome assembly software have become more affordable and accessible [1]. Current technologies are often grouped into second- and third-generation sequencing [2]: second-generation sequencing technologies are dominated by Illumina platforms and are characterized by short (up to a few hundred base pairs) but highly accurate reads that can be produced in millions at low cost [2–4]. The disadvantage of this technology is that short reads are often smaller than repetitive regions in bacterial genomes. Therefore, many genome assemblies based on short reads alone are incomplete and fragmented into multiple continuous sequences, so-called contigs [5–7].

Third-generation sequencing, such as Oxford Nanopore Technology (ONT), produces much longer reads (up to hundreds of thousands of base pairs), which are more likely to span large repeat regions, allowing a complete genome reconstruction [8, 9]. However, long reads are less accurate [10] and must be corrected by either (i) long-read self-correction or (ii) a hybrid approach using a combination of long- and short-read data [11]. Long-read assemblers, such as Flye [12], Canu [13] and Raven [14], reconstruct bacterial genomes solely from long sequencing reads, and it has been repeatedly demonstrated that these assemblies are of high quality. However, long-read genome assemblers often struggle to assemble plasmid sequences and are prone to missing small plasmids [8, 15–17]. Therefore, a recent study suggested using the hybrid assembler Unicycler, when plasmid recovery is important [16].

Unicycler uses both short (Illumina) and long reads (Oxford Nanopore or PacBio), following a short-read first hybrid approach [7]. It first assembles short reads into an assembly graph, which is a data structure containing both (i) contigs and (ii) their possible connections to other contigs. Then, long reads are added to resolve the graph. Unicycler is available on public Galaxy instances, which allow even non-expert users to perform genome assemblies through a simple web interface. Beginners can easily learn how to pre-process raw sequencing data and start an assembly by following tutorials on the Galaxy Training Network (https://training.galaxyproject.org/). Ideally, the automatic assembly with Unicycler leads to a completely resolved genome, i.e. each replicon (chromosome or plasmid) should be represented by a single contig [7]. If the fully automated assembly process cannot resolve a bacterial genome, manual editing is required to complete it [18, 19]. As there is no standardized, straightforward process, manual genome completion depends highly on a researcher’s experience and may be challenging. However, the Unicycler webpage provides examples and helpful tips for finishing genomes (https://github.com/rrwick/Unicycler/wiki/Tips-for-finishing-genomes).

According to our experience working with insect-associated members of the *Bacillus cereus* group, mainly two mistakes may occur in the process of Uniclyer’s hybrid assembly: (i) collapse of large inter-plasmidic repeats and (ii) removal of genuine single-copy contigs. An inter-plasmidic repeat refers to an identical or highly similar sequence shared by multiple plasmids [20]. Repetitive regions are difficult to assemble, because the assembly graph then does not contain a single distinct path for the underlying sequences. This may result in an incorrect number and location of repeat sequences in the final assembly, so-called collapsing repeats [21, 22]. For example, genomic regions that are not biologically continuous (e.g. two different plasmids) may be artificially fused (‘falsely joined’) by the assembly algorithm [18, 23, 24]. Besides the difficulties in resolving large inter-plasmidic repeats, we found that Unicycler sometimes removes genuine single-copy contigs. These single-copy contigs occur once in the underlying genome sequence [7], but are absent from the final hybrid assembly.

Both of the two errors described are caused by mistakes in Unicycler’s contig multiplicity determination. Multiplicity refers to how many times a particular contig appears in the genome. Following the short-read assembly, Unicylcer needs to (i) distinguish between single-copy (multiplicity = 1) and repeat contigs (multiplicity > 1) and (ii) specify the correct multiplicity of repeat contigs [7]. If Unicycler does not determine contig multiplicity properly, the genome is not being assembled correctly. These assembly errors have negative effects on downstream analysis, since generating assemblies is often the first step in a bioinformatics pipeline [25].

In this study, we propose a Galaxy-based workflow for manual genome completion, addressing two mistakes, which may occur in Unicycler’s automatic assembly. This workflow requires both Illumina and Nanopore sequencing data of the same bacterial strain, but does not demand expertise in programming or running programs from the command line. By applying the proposed standardized approach, researchers might be encouraged to evaluate their auto-assemblies carefully and decipher the chromosome and plasmid composition of a genome sequence in a more accurate way than simple automatic procedures can achieve.

## Methods

### Bacterial strains and culture conditions

Two species of the *Bacillus cereus* group were chosen to demonstrate two mistakes that may occur in the process of whole genome assembly using Unicycler (Table 1). First, *Bacillus thuringiensis* subsp. *tenebrionis* (Btt) strain ‘ten BI 256-82/CM3’ (JKI core database strain notation; ten = tenebrionis) was deposited in the JKI microbial collection in 1990. Second, a spore-forming *Bacillus wiedmannii* (Bw) bacterium, termed strain EPS29, was isolated from a field-collected third-instar oak processionary moth larva in 2018. Bacterial strains were long-termed stored at −80 °C in glycerol (BI256/CM3) or Microbank vials (EPS29). For recovery, strains were streaked onto LB agar plates and grown at 30 °C. Five millilitres of liquid LB medium (1 % tryptone, 0.5 % yeast extract, 0.5 % NaCl, pH 7.0±0.2; Carl Roth) was inoculated with a single colony and incubated at 30 °C with shaking at 200 r.p.m. overnight.

**Table 1.** *Bacillus* sp. strains used for whole genome sequencing

Strain notation	Species	Origin	Deposit date*
ten BI 256-82/CM3	*Bacillus thuringiensis* subsp. *tenebrionis*	*Tenebrio molitor*	27 June 1990
EPS29	*Bacillus wiedmannii*	*Thaumetopoea processionea*	7 May 2018

*Both strains were obtained from the microbial collection at the Julius Kühn-Institut (JKI), Institute for Biological Control, Dossenheim, Germany.

### Genomic DNA extraction, quality control and sequencing

Total genomic (chromosomal and plasmid) DNA (gDNA) was extracted from 1 ml of overnight culture using the Wizard Genomic DNA Purification Kit (Promega), following the protocol for Gram-positive bacteria. However, any kit extracting both chromosomal and plasmid DNA may be used, provided that the quality (no degradation, OD ratios 260/280 ≈ 1.8 and 260/230 = 2.0–2.2) of high-molecular-weight genomic DNA is sufficient. Short-read sequencing was conducted on an Illumina NextSeq 2000 sequencer (StarSEQ) using a 150 bp paired-end protocol. Long-read sequencing was performed on a MinION R9.4.1 flowcell using the MinION sequencer (Oxford Nanopore Technologies). If necessary, extracted genomic DNA was concentrated to approximately 30 ng µl^–1^ prior to library preparation using an Eppendorf Concentrator plus (Eppendorf). Multiplexing and library construction were performed using the Native Barcoding Expansion Kit (EXP-NBD104) and Ligation Sequencing Kit (SQK-LSK109), following the manufacturers’ instructions with minor modifications: for DNA washing, 80 % ethanol was used. The time for gDNA binding to and elution from magnetic beads was increased to 15 min. To enrich for long DNA fragments the Long Fragment Buffer (LFB), provided by the kit, was used and the final elution step was conducted at 37 °C. After finishing the sequencing run, basecalling of ONT reads (min quality score = 7) was performed using a high-accuracy basecalling model with Guppy v4.3.4 software. Following the basecalling, reads were demultiplexed and adapter trimmed locally using Guppy v4.3.4.

**Table 2.** Bioinformatics tools that were used to assemble and complete the entire bacterial genomes (chromosome and plasmids)

Tool	Purpose	Operating system	Run on	Reference
Concatenate datasets tail-to-head (cat) (Galaxy Version 0.1.1)	Reads pre-processing	Linux	Galaxy instance	
FastQC (Galaxy Version 0.72+galaxy1)	Short reads quality contol	Linux	Galaxy instance	[28]
Trim Galore! (Galaxy Version 0.6.3)	Short reads pre-processing	Linux	Galaxy instance	[29]
NanoStat (Galaxy Version 0.1.0)	Long reads quality control	Linux	Galaxy instance	[30]
NanoFilt (Galaxy Version 0.1.0)	Long reads pre-processing	Linux	Galaxy instance	[30]
Unicycler (Galaxy Version 0.4.8.0)	Genome assembly	Linux	Galaxy instance	[7]
Bandage (v0.8.1.)	Genome assembly evaluation	Windows 10	Local computer	[26]
BUSCO (Galaxy Version 5.2.2+galaxy0)	Genome assembly evaluation	Linux	Galaxy instance	[31]
Compute sequence length (Galaxy Version 1.0.3)	Genome assembly evaluation	Linux	Galaxy instance	[32]
BWA-MEM (Galaxy Version 0.7.17.2)	Genome assembly evaluation	Linux	Galaxy instance	[36, 37]
Samtools stats (Galaxy Version 2.0.2+galaxy2)	Genome assembly evaluation	Linux	Galaxy instance	
Geneious prime (v2021.2.2)	Genome assembly evaluation	Windows 10	Local computer	Biomatters
Text editor	Modification of GFF3 files (multiplicity adjustments)	Windows 10	Local computer	e.g. EditPad, Windows Notepad

### Software for bioinformatics analysis

To pre-process sequencing data and assemble genomes, different bioinformatics tools were required (Table 2). Some tools are only available for Linux-based operating systems and have an official implementation for Galaxy instances. The tools may also be available on other operating systems, which should not change the outcome of the analysis. Most of the bioinformatics analysis was performed on a Galaxy instance at the Julius Kühn Institute (JKI). To allow manual adjustments, the option ‘re-run Unicycler using modified best SPAdes graph as input’ was implemented in the local Galaxy server. The Galaxy tools used in this work are also available on public instances of Galaxy (e.g. https://usegalaxy.org, https://usegalaxy.eu). Alternatively, any analysis done in Galaxy can be run locally from the command line. Besides the Galaxy tools, some software programs were used in Windows 10: the software Bandage (v0.8.1.) [26] was downloaded from the webpage https://rrwick.github.io/Bandage/. To use Bandage’s built-in blast search, NCBI blast was downloaded (https://ftp.ncbi.nlm.nih.gov/blast/executables/blast+/LATEST/) and installed directly into the Bandage folder. A text editor for Windows, such as EditPad or Windows Notepad, was used to open and modify GFF3 files. To view read alignments and extract ambiguous regions, the software Geneious Prime (v2021.2.2) (Biomatters) was utilized, but any open-source genome viewer [e.g. Integrative Genomics Viewer (IGV) https://software.broadinstitute.org/software/igv/] [27] may be used alternatively. To visualize the collapsed repeat region, the software R (v4.1.2.) using RStudio (v2021.09.2) was used, though R is not required for the proposed workflow. The R script, which was used to create Figs 4 and S1, is available in the online version of this article (Supplementary Material 1).

### Quality control and preprocessing of short-read sequencing data

**Fig. 1.**
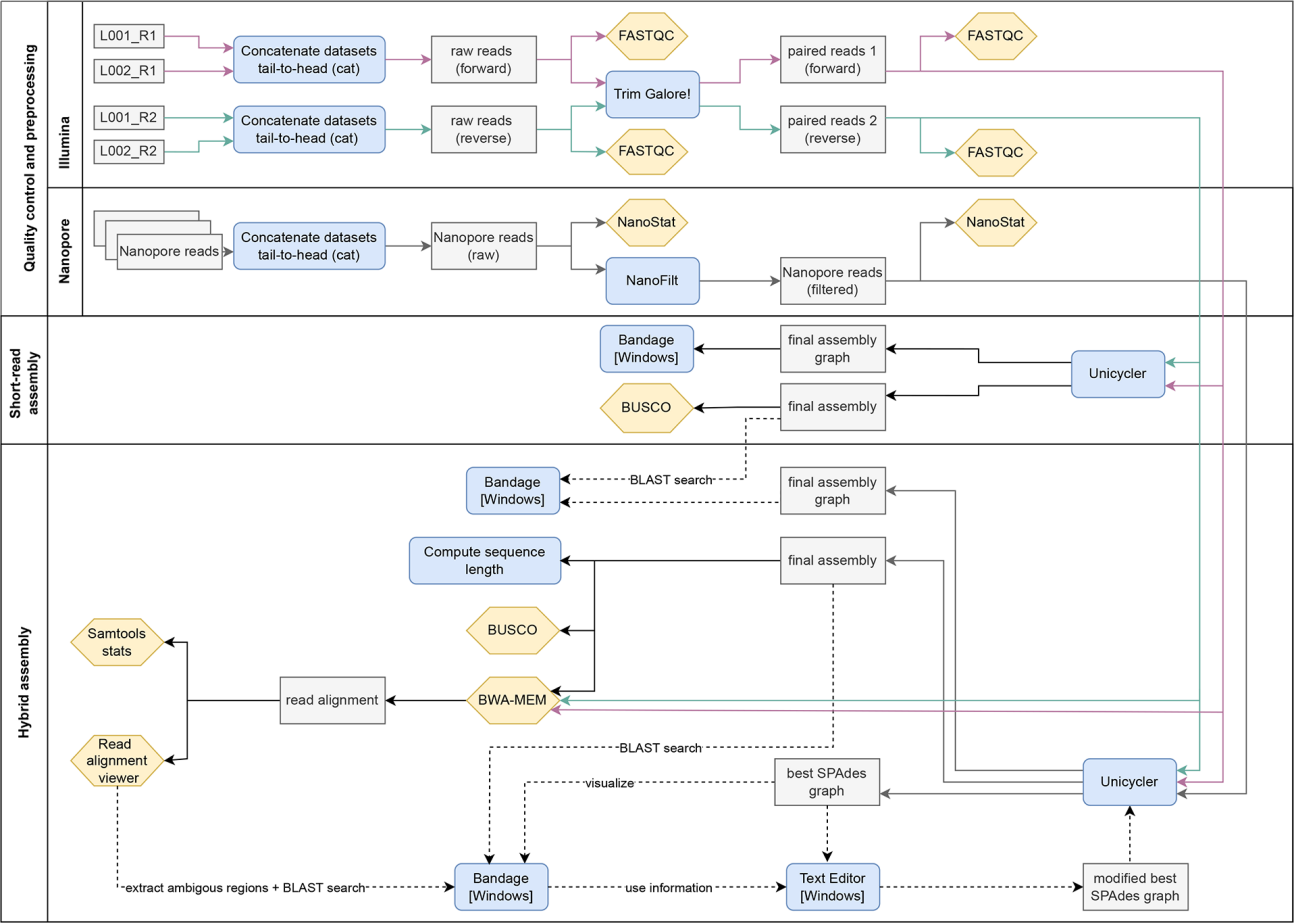
Workflow for bacterial genome assembly. Optional steps for manual genome completion are displayed as dotted lines.

For the Illumina paired-end sequencing data one R1 (forward reads) and one R2 (reverse reads) FASTQ file was created for each sample for each lane. Compressed FASTQ files (fastq.gz) were uploaded on the JKI Galaxy server. If the sample was run on multiple lanes, FASTQ files were concatenated to produce a single FASTQ file containing raw forward reads and a single FASTQ file containing raw reverse reads (Fig. 1). The quality of raw Illumina read data was checked with FastQC (v0.72) [28]. Subsequently, raw reads were adapter trimmed and quality filtered (Phred quality score ≥30; minimum read length after trimming =20 nt, output paired-end reads only) using Trim Galore! (v0.6.3.) [29], followed by quality re-assessment with FastQC (Fig. 1). Illumina paired-end sequencing produced a total of 6.3 million (ten BI 256-82/CM3) and 9.6 million (EPS29) reads with an average Phred quality score of Q = 33 (Table 3). The number of reads decreased by 1.4 % (ten BI 256-82/CM3) and 0.05 % (EPS29) after adapter removal, quality trimming and read pairing (Table 3).

**Table 3.** Sequencing summary table for *B. thuringiensis* subsp. *tenebrionis* strain ten BI 256-82/CM3 and *B. wiedmannii* strain EPS29

Strain	Short read sequencing (Illumina)		Long read sequencing (MinION)		
	No. of total reads	No. of quality filtered reads*	No. of total reads†	No. of quality and length filtered reads‡	N50 (nt)
ten BI 256-82/CM3	6 256 887	6 168 013	1 514 538 (A)	1 014 539	7536
EPS29	9 583 524	9 578 788	4 308 301 (B)	2 615 696	2733

*TrimGalore!: adapter removal and quality trimming (Phred quality score ≥30; minimum read length after trimming = 20 nt), output of paired reads only.

†Different letters indicate that samples were run on different flowcells.

‡Nanofilt: minimum read quality Q=10, minimum read length = 1000 nt.

### Quality control and preprocessing of long-read sequencing data

Multiple compressed FASTQ files (fastq.gz) generated after basecalling were uploaded on the JKI Galaxy server. To organize the uploaded files in a single dataset collection, the *Build Dataset List* function was used. Files within the dataset collection were concatenated (Concatenate datasets tail-to-head; cat) (Galaxy Version 0.1.1) to obtain a single file containing all the raw ONT reads (Fig. 1). In Galaxy, the fastqsanger format, which is a datatype for FASTQ files with Phred33 quality score encoding, is needed to run many tools. Since our dataset was already in the required format, the datatype format was simply changed to fastqsanger.gz using the pencil icon. Both long ONT read data sets were filtered on minimum read quality (Q = 10) and minimum read length (≥1000 bp) using NanoFilt (v2.3.0) [30]. For each sample, basic statistics of long-read sequencing data before and after filtering were calculated with NanoStat (v1.1.2.) [30]. Long-read data belonging to Btt strain 'ten BI 256-82/CM3' was additionally filtered on a minimum read length of 10 000 bp. These two different length-filtering criteria (≥1000 bp, ≥10 000 bp) were applied, because in this particular case it caused the collapsed repeat to look different. This allows us to demonstrate how two distinct appearances of a collapsed repeat region can be detected and resolved. Long-read sequencing (ONT) generated a total of 1.5 million (ten BI 256-82/CM3) and 4.3 million (EPS29) reads with an N50 read length of 7.5 kb (ten BI 256-82/CM3) and 2.7 kb (EPS29). After quality (Q = 10) and length (≥ 1000 bp) filtering, the number of reads was reduced by 33 % (ten BI 256-82/CM3) and 39 % (EPS29) (Table 3).

### Short-read-only assembly

For an initial genome assembly, the paired-end and quality filtered Illumina short reads were assembled with Unicycler (default parameters, normal bridging mode) [7]. This first assembly was performed to (i) evaluate the short-read dataset, (ii) check for small plasmids and (iii) detect false gene losses in a hybrid assembly. In Galaxy, it produces two outputs: the final short-read only assembly in FASTA format and the final assembly graph in graph format (gfa1) (Fig. 1). The final assembly contains a list of contiguous DNA sequences (contigs), whereas the final assembly graph also shows possible connections between these contigs [26]. To assess whether the short-read dataset is suitable for a hybrid approach with Unicycler, the final short-read assembly graph was loaded in Bandage (Fig. 1). As a short-read first assembler, Unicycler requires a high-quality short-read assembly graph. With default settings, Unicycler creates ten different short-read assembly graphs and automatically selects the best one by minimizing both contig counts and dead-ends [7]. The term dead-end describes that a contig end is not linked to any other contig in the assembly graph. If the short-read depth is too low, the graph is fragmented, i.e. contains many dead-ends. This prevents Unicycler from resolving the graph in the later step of the pipeline. Therefore, the short-read graph should have as few dead-ends as possible. To assess the quality of the short-read assembly in terms of expected gene content, BUSCO (v5.2.2.; genome mode) [31] with lineage-specific orthologue set for *Bacillales* containing 450 core genes was used (bacillales_odb10, 2021-02-23) (Fig. 1).

### Hybrid auto-assembly and evaluation

After reviewing the short-read-only assembly and being satisfied with the quality, the hybrid assembly was conducted with Unicycler (default parameters, normal bridging mode) using the paired-end short (Illumina) and long (ONT) sequencing data (Fig. 1). Overall, three criteria were used to evaluate the quality of the hybrid auto-assembly: (i) contig circularity, (ii) BUSCO-completeness and (iii) remapping of reads.

Contig circularity: To get a quick overview about the output of the hybrid auto-assembly, the tool Compute sequence length (Galaxy Version 1.0.3) [32] was used (Fig. 1). It produces a tabular output file listing all contigs with their length in descending order and displaying the circularity of contigs by adding the specification ‘circular = true’. In a fully resolved bacterial genome, each replicon (i.e. chromosome/plasmid) should be represented by a single contig. As bacterial replicons are usually circular [17], Unicycler considers a circular contig to be complete [7]. Even though linear chromosomes and plasmids appear to be rare in bacteria, they have been reported for some species [33–35]. In this case, a linear replicon would be fully represented by a linear contig. However, this special case is not discussed further in this workflow and would have to be analysed separately.

BUSCO completeness: BUSCO (v5.2.2.; genome mode) was run on the hybrid auto-assembly to search for the presence of 450 *Bacillales* core genes (Fig. 1). In general, it is recommended to compare the BUSCO scores of various assemblies (e.g. Illumina-only, hybrid auto-assembly, final hybrid assembly) in the process of whole genome reconstruction.

Remapping of reads: Short reads were mapped back to the assembled genome using BWA-MEM [36, 37] and the percentage of mapped reads was determined using Samtools stats. Ideally, a vast majority of reads should align to their respective assembly. Coverage and depth of mapping were visually inspected in Geneious Prime (v2021.2.2; Biomatters), but any other genome viewer (e.g. IGV) can be used alternatively.

### Manual completion

**Fig. 2.**
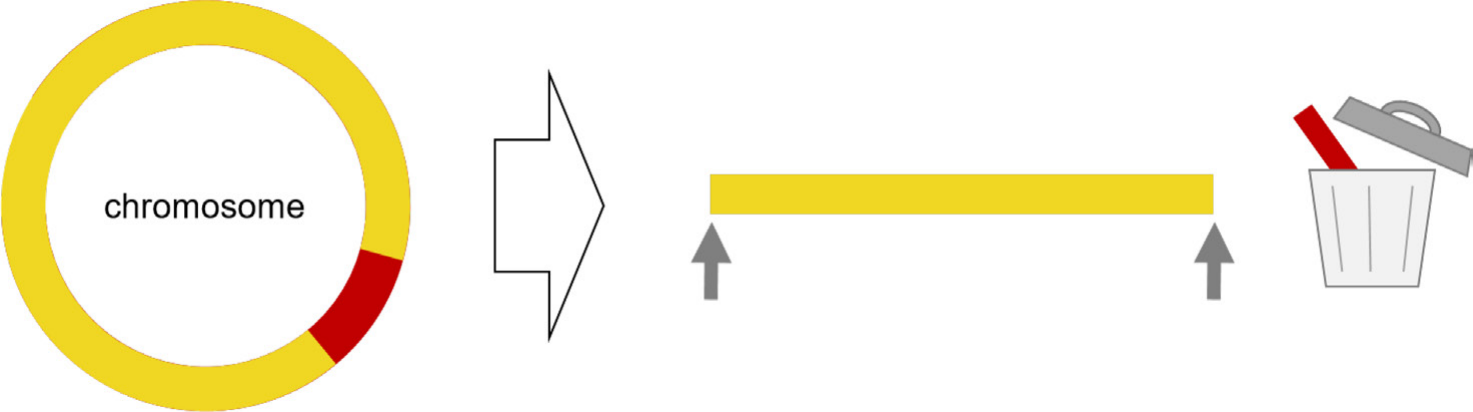
False loss of single-copy sequences. If genuine single-copy contigs are mistakenly removed during genome assembly, this will result in dead-ends in the final assembly graph (marked with grey arrows).

**Fig. 3.**
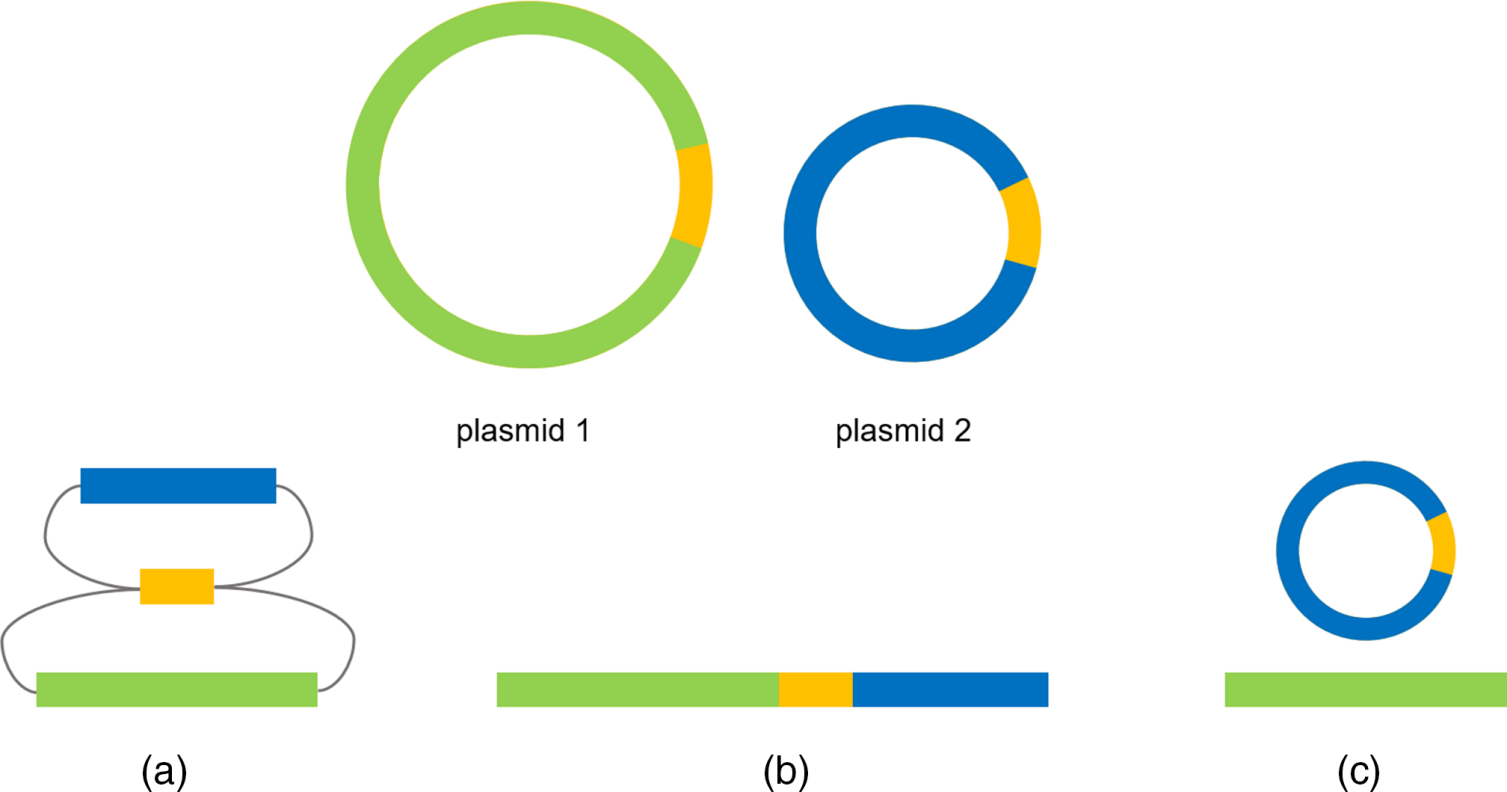
Large repeat regions shared by distinct plasmids often collapse during the assembly process. The shared repeat is shown in orange and the unique parts are shown in green (plasmid 1) and blue (plasmid 2), respectively. A single collapsed repeat region can lead to three different results in the hybrid assembly. (**a**) The two circular plasmids are represented by a total of three linear contigs. Each of the two unique parts are connected to the repeat contig in the assembly graph. (**b**) The two plasmid contigs are falsely joined due to the collapsed repeat, resulting in a single linear contig. (**c**) The repeat sequence is assigned to only one plasmid, resulting in one circular plasmid and one linear plasmid contig.

If the genome is not completely resolved, the three quality control steps described above help to detect mistakes in the hybrid auto-assembly and finish the genome with some manual editing. First, the final assembly graph was visualized in Bandage to revisit the problematic parts of the hybrid auto-assembly (Fig. 1). In general, dead-ends (i.e. no possible connections to any other contigs) in a hybrid assembly graph often indicate missing sequences (Figs 2 and 3). This does not necessarily have to be caused by a collapsing repeat region (Fig. 3), but genuine single-copy contigs may also be lost during the assembly process (Fig. 2). In both cases, the multiplicity calls of the problematic regions should be viewed in Bandage using Unicycler’s graph custom colour scheme (green = single copy, yellow = two copy, orange = three copy, red = four or more copies), followed by manual multiplicity adjustments if necessary.

Detailed instructions on how to adjust these multiplicities can be found on the Unicycler GitHub page (https://github.com/rrwick/Unicycler#manual-multiplicity).

### False loss of single-copy contigs

To avoid false gene losses due to missing single-copy contigs (Fig. 2), we recommend searching the short-read only (Illumina) assembly (fasta) in the hybrid auto-assembly graph using the software Bandage and its integrated blast search (Fig. 1). If a large contig of the short-read only (Illumina) assembly is absent from the hybrid assembly, Unicycler’s multiplicity call of that contig should be inspected in the best SPAdes graph (Fig. 1). If there is no multiplicity call (the contig appears grey), the multiplicity should be manually added. Briefly, the best SPAdes graph (in gfa1 format) was opened in a text editor and the respective GFA segment line was searched using the specific contig name (SX, where X is the individual name, i.e. a number, of each contig). After setting a tab stop to the end of the respective GFA line, the contig’s multiplicity was specified by adding an ML tag (ML:i:1 for single-copy contigs). The modified best SPAdes graph was then used to re-run Unicycler (Fig. 1).

### Collapsing inter-plasmidic repeats

The approach used to identify missing single-copy sequences (see previous paragraph) is not suitable for identifying collapsing repetitive regions. In our experience, a collapsed repeat region can lead to three different results in the final assembly graph:

(A) A single repeat region appears to be shared by two circular contigs. Final assembly graphs may reveal unresolved repeats (Fig. 3a), which can be easily resolved in Bandage using the graph editing functions (*Duplicate selected nodes*, *Remove selection from graphs*, *Merge selected nodes*). However, it is often not possible to identify large collapsing repeat regions from the assembly graph (B, C).

(B) False join of two plasmid contigs to a linear unresolved contig. A large repeat region shared by two plasmids may be algorithmically collapsed into a single sequence. This may cause two plasmid contigs to be falsely joined, creating a large linear contig with two dead-ends (Fig. 3b).

(C) Excision of repeat region from one but not the other plasmid. Alternatively, the collapsing repeat sequence may be assigned to one plasmid contig, which makes one plasmid look circular and the other look incorrectly linear, because there is no sequence connecting the two ends (Fig. 3c).

In the latter two cases (B, C), re-mapping of reads to the final hybrid auto-assembly (as above) can be used to detect problematic areas. If the assembler mistakenly merges two distinct contigs (B, Fig. 3b), this can be detected by an excessive read depth within the collapsed repeat sequence [21]. The identified sequence was extracted (copy collapsed repeat region) and subsequently blast searched in the best SPAdes graph using Bandage (Fig. 1). Subsequently, multiplicities of contigs in the identified repeat region were viewed and adjusted if needed. To reduce the number of manual multiplicity checks, only contigs ≥1000 bp were considered for visual inspection. Then, Unicycler was re-run using the modified best SPAdes graph as input (Fig. 1).

A linear contig with a uniform read depth (C) should be checked to see if a sequence is missing that would make the contig circular. Here, it is recommended to focus on the two ends of the linear contig: the start (1000 bp) and end (1000 bp) of the linear contig were copied and saved as a FASTA file. To determine if the linear contig has possible connections to other contigs, the two extracted sequences were blast searched in the best SPAdes graph. Displaying the blast hits in rainbow colour allows viewing the direction of the sequence and possible connections to other contigs may be identified. By searching the final hybrid auto-assembly in the best SPAdes graph, it can be detected to which contig/replicon there is a possible connection. Subsequently, the read alignment of this identified contig/replicon should be viewed to detect a possible collapsed sequence by excessive read depth. The subsequent steps are the same as described in the previous paragraph (B).

### Counting occurrences of errors

To estimate the occurrence frequency of repeat collapse and false loss of single-copy contigs, sequencing data (Illumina+Nanopore) were downloaded from NCBI SRA and assembled using Unicycler. Bacterial strains were chosen that are known to contain (i) multiple plasmids and (ii) repetitive genomic regions. Eleven additional *Bacillus thuringiensis* strains (SAMN31131915, SAMN37105265, SAMN37105266, SAMN31131916, SAMN31131917, SAMN21214336, SAMN21214335, SAMN21214334, SAMN21214333, SAMN21214332, SAMN21214338) and seven clinically relevant strains including two *Klebsiella* (SAMN07211282, SAMN07211281), two *Enterobacter* (SAMN10174734, SAMN36773911) and three *Citrobacter* (SAMEA5578852, SAMEA5578849, SAMEA5578862) strains (Table S1) were analysed.

## Results and discussion

To decipher complex bacterial genomes, a Galaxy and user-friendly workflow was established that combines the strengths of both accurate short-read (Illumina) and long-read (Nanopore) sequencing. The workflow was divided into three main sections: (i) quality control and preprocessing, (ii) short-read assembly and (iii) hybrid assembly. After read filtering and quality control, the Illumina reads were used for a short-read only assembly to initiate a first quality and completeness check based on the assembly graph and BUSCO score. Subsequently, a hybrid assembly based on both short- and long-read data was conducted. If the genome was not fully resolved (presence of dead-ends in hybrid assembly), problems were solved retroactively to the assembly by correcting multiplicities in the best SPAdes graph. The modified SPAdes graph was then used to restart the hybrid assembly. This process can be repeated several times until the genome is completely resolved.

For strains ‘ten BI 256-82/CM3’ and Bw EPS29 only one additional round with adjusted multiplicities was required to resolve their genomes entirely. To confirm that collapsing repeat regions also occur in other bacterial auto-assemblies, 11 additional *B. thuringiensis* strains with available Illumina and Nanopore data were assembled and in seven assemblies collapsing repeat regions were observed. To extend the analysis beyond biopesticide strains, seven clinically relevant bacterial strains of the genera *Klebsiella*, *Enterobacter* and *Citrobacter* were included in the analysis, where the issue of repeat collapse occurred in six out of seven cases. False loss of large single-copy contigs could only be found in strain Bw EPS29 of the present study.

### Collapse of inter-plasmidic repeats

In general, the hybrid assembly pipeline Unicycler is able to resolve many repeats through the combination of short- and long-read sequencing data. However, Unicycler appears to have difficulties resolving large inter-plasmidic repeats and additional editing is required to finish these genomes.

The final genome assembly of Btt strain ‘ten BI 256-82/CM3’ consisted of one circular chromosome (5.6 Mb, GC content = 35.3 %) and five circular plasmids ranging in size from 14.9 to 250.5 kb and GC content from 30.4 to 33.9 % (Table 4). The BUSCO completeness score was 99.8 % (S: 98.9 %, D: 0.9 %, F: 0.0 %, M: 0.2 %, N: 450) and 99.96 % of short reads mapped back to the assembly.

**Table 4.** Final genome assembly of *B. thuringiensis* subsp. *tenebrionis* strain ten BI 256-82/CM3

Replicon	Length (bp)	GC (%)	Topology
Chromosome	5 619 078	35.3	Circular
ppl250	250 492	33.9	Circular
ppl185	185 418	32.6	Circular
ppl77	77 374	30.4	Circular
ppl68	68 504	32.2	Circular
ppl14	14 853	31.0	Circular

The two largest plasmids (ppl250 and ppl185) of the Btt genome (Table 4) exhibit a 47.7 kb inter-plasmidic repeat, making it initially challenging to assemble them. To demonstrate two distinct appearances of this collapsed repeat, two auto-assemblies (Table 5, A and B) were performed, employing different ONT length-filtering criteria [≥10 000 bp (A) or ≥1000 bp (B)]. Using ONT reads that were at least 10 000 bp long resulted in a single falsely joined contig with a total size of 2A = 387.3 kb (Table 5, A). If ONT reads with a minimum length of 1000 bp were used, it was possible to completely resolve the 3B = 185.4 kb plasmid (Table 5, B) However, the other plasmid was incomplete and appeared to be a linear contig of 2B = 201.9 kb (Table 5, B).

**Table 5.** Output of hybrid auto-assemblies of *B. thuringiensis* subsp. *tenebrionis* strain ten BI 256-82/CM3 Auto-assembly was performed twice with ONT reads filtered by a minimum read length of ≥10 000 bp (A) or ≥1000 bp (B). Different length filtering was used to demonstrate two different possible outputs of an algorithmically collapsed repeat region: collapsing repeat regions may result in a single falsely joined contig (2A) or in one complete (circular) contig (3B) and a linear, incomplete contig due to the missing sequence (2B)

**A**			**B**		

Contig	Length (bp)	Topology	Contig	Length (bp)	Topology
1A	5 619 037	Circular	1B	5 619 078	Circular
2A	387 316	Linear	2B	201 897	Linear
			3B	185 418	Circular
3A	77 374	Circular	4B	77 374	Circular
4A	68 504	Circular	5B	68 504	Circular
5A	14 853	Circular	6B	14 853	Circular

### Hybrid auto-assembly A (ONT read length ≥10 000 bp)

**Fig. 4.**
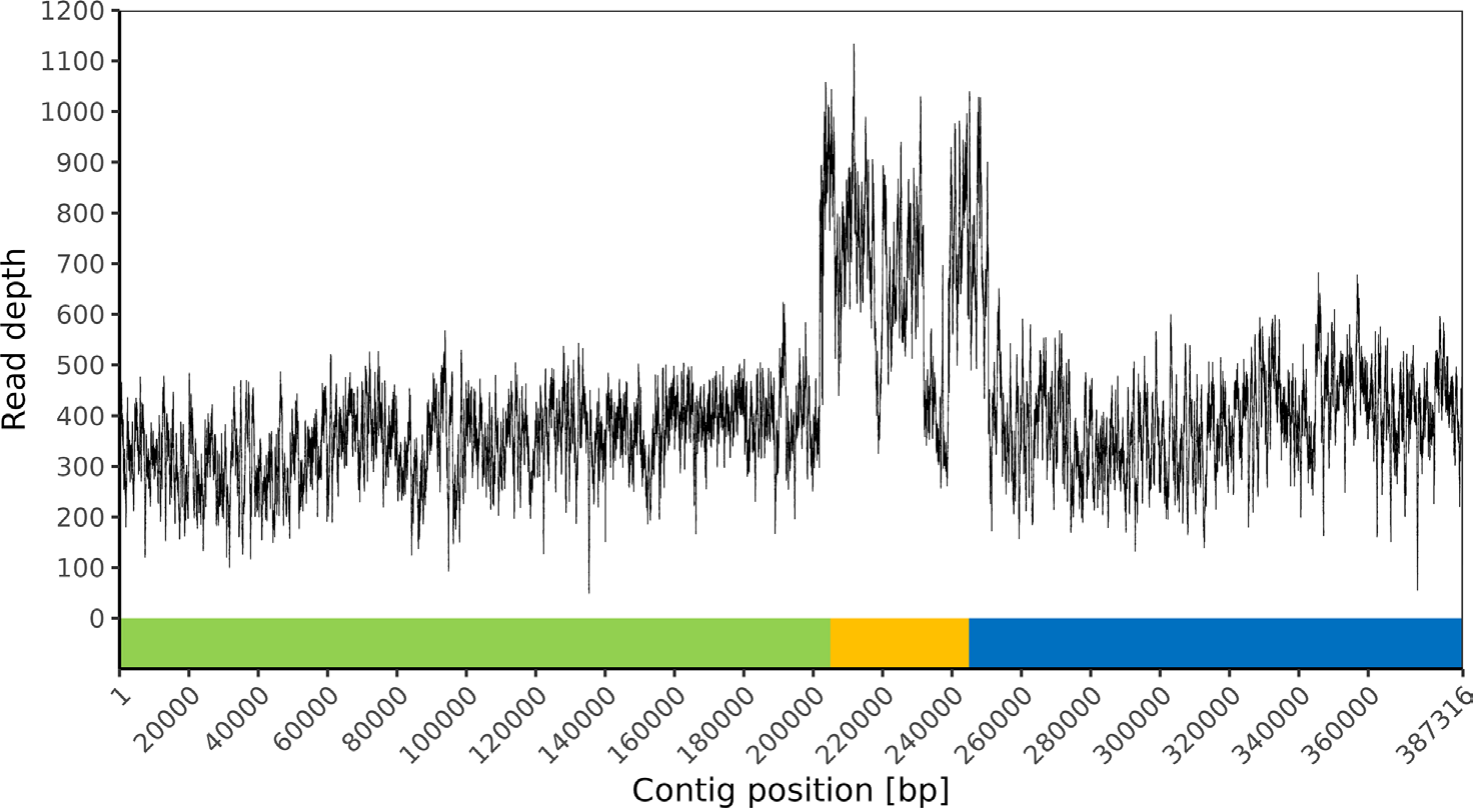
An excessive read depth indicates the collapsed repeat region in the linear contig 2A = 387.3 kb (Table 5). Short reads of *B. thuringiensis* subsp. *tenebrionis* strain ten BI 256-82/CM3 were re-mapped against the respective auto-assembly. The collapsed inter-plasmidic repeat is marked in orange and the non-repeat parts of the 250.5 kb plasmid and 185.4 kb plasmid are shown in green and blue, respectively.

**Fig. 5.**
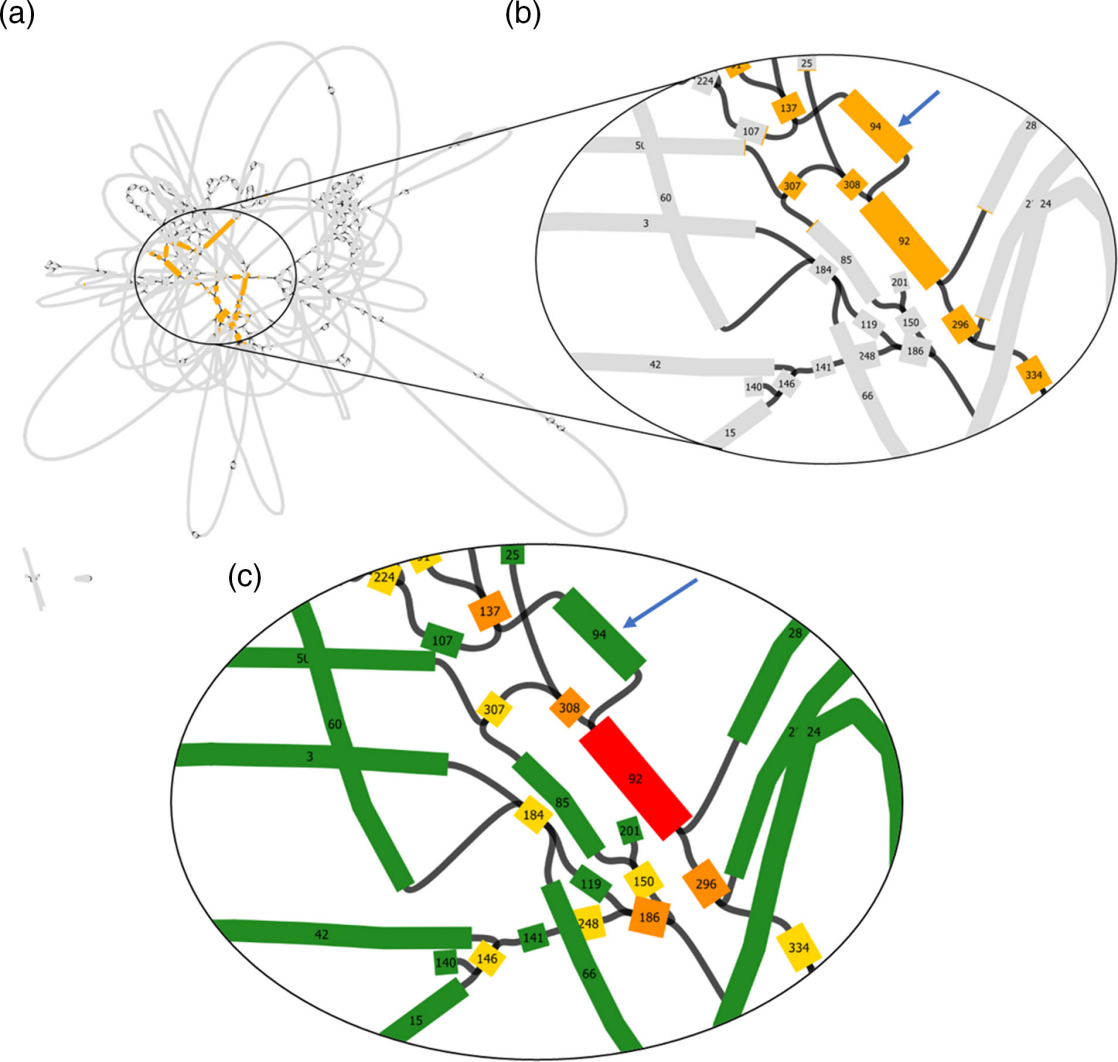
Bandage visualization of the best SPAdes graph of hybrid auto-assembly A (ONT read length ≥10 000 bp). Contigs are represented by boxes and contig connections are visualized by black lines. The collapsed repeat sequence previously identified by read back mapping was blast searched in Bandage. The blast hits were visualized in orange using Bandage’s solid colour scheme (**a, b**). Numbers within boxes indicate contig ID as assigned by Unicycler after short-read assembly. Changing the colour scheme to custom colours allows us to view Unicylcer’s multiplicity calls (green = single copy, yellow = two copy, orange = three copy, red = four or more copies). The multiplicity of contigs that are at least 1000 bp in length and belong to the identified repeat region was checked. Contig 94 (marked with a blue arrow, length = 5219 bp) belonging to the inter-plasmidic repeat appears green in the best SPAdes graph (**c**), meaning it was mistakenly considered single-copy by Unicycler.

Back-mapping of short reads against the hybrid auto-assembly revealed the collapsed repeat region within the falsely joined contig by excessive read depth (Fig. 4). The identified sequence was extracted and subsequently blast searched in the best SPAdes graph of the hybrid auto-assembly (Fig. 5). In most cases, Unicycler correctly assigned the multiplicities of contigs belonging to the inter-plasmidic repeat. A single contig (contig name in best SPAdes graph = 94, length = 5219 bp) was mistakenly considered single-copy (Fig. 5), leading to the algorithmic collapse of the entire 47.7 kb repeat sequence.

After manually indicating a multiplicity of 2 for the specific contig by adding an ML tag (ML:i:2) to the end of the respective GFF line (S 94), Unicycler was restarted using the modified best SPAdes graph as input (Fig. 1). Taking into account the corrected multiplicity Unicycler was able to separate the two plasmids and fully resolve the genome (Table 4).

### Hybrid auto-assembly B (ONT read length ≥1000 bp)

Back-mapping short reads against hybrid auto-assembly B revealed a relatively uniform read depth across the linear contig 2B (Table 5), which makes a collapsed sequence within the contig seem unlikely. However, the presence of reads that extend beyond the borders of the linear contig (but do not encompass both contig ends) indicates a missing sequence.

**Fig. 6.**
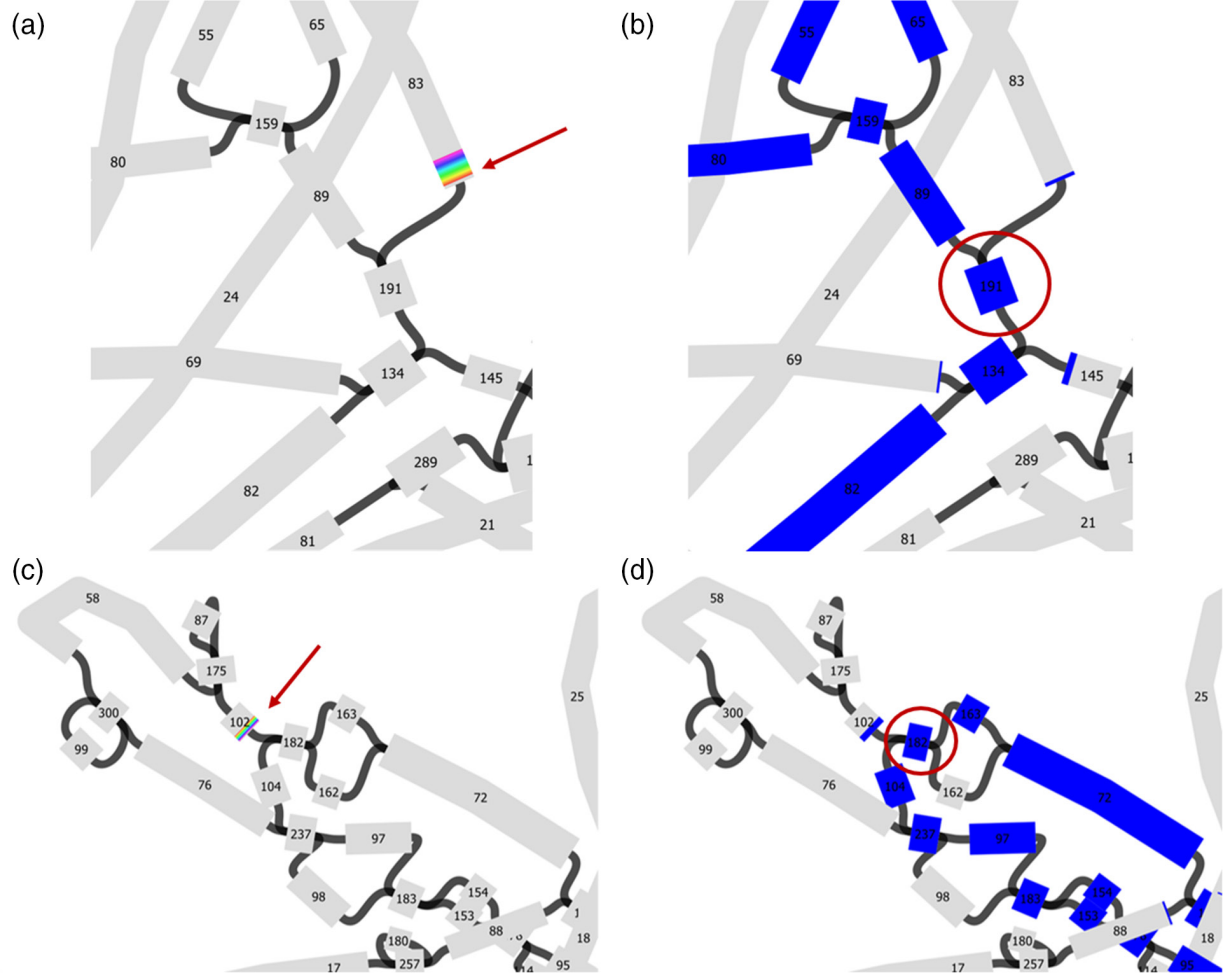
Close-up Bandage visualization of the best SPAdes graph of hybrid auto-assembly B (ONT read length ≥1000 bp). Contigs are represented by boxes and contig connections are visualized by black lines. Numbers within boxes indicate contig ID as assigned by Unicycler after short-read assembly. To determine if the 201.9 kb linear contig 2B (Table 5) has possible connections to any other replicon, 1000 bp from both sides of the linear sequence were blast searched in the best SPAdes graph using Bandage. Displaying the blast hits in rainbow colours (**a, c**) allows us to view the direction of the sequence. Both ends of the linear contig 2B are linked to the 185.4 kb plasmid (displayed in blue, **b, d**).

To investigate whether the missing sequence resulted from a collapsed repeat, we focused on the two contig ends: the first and last 1000 bp of the linear sequence (contig 2B) were extracted and blast searched in the best SPAdes graph to check for possible connections to any other contigs. According to the best SPAdes graph, the linear contig 2B is linked to contigs belonging to the circular 185.4 kb plasmid contig 3B (Fig. 6). Hence, reads mapping to plasmid contig 3B were visually examined and an excessive read depth on part of the sequence indicated a repeat region (Fig. S1). The identified sequence was extracted and blast searched in the best SPAdes graph. The multiplicity of three contigs (S 82, S 94, S 134) belonging to the inter-plasmidic repeat were corrected (Table S2) and Uniclycler was run again, leading to a fully resolved genome.

Our long-read dataset contains reads that are long enough to span the entire 47.7 kb inter-plasmidic repeat and be anchored in the unique sequences on both sides of the repeat region [25]. Despite this evidence in the long-read data, a small number of multiplicity mistakes had detrimental effects on the rest of the automatic assembly. Manually correcting a few (one to three) contig multiplicities prevented the inter-plasmidic repeat from collapsing and resulted in a fully resolved genome. Each replicon, including the two largest plasmids (ppl250, ppl185), is finally presented by a single circular contig (Table 4). The 47.7 kb repeat region, which incorrectly appeared only once in both auto-assemblies, is present twice in the final assembly and is located once on the circular plasmid ppl250 and once on the circular plasmid ppl185.

In general, the assembly of plasmid sequences from genomic data is not trivial and often results in (i) incomplete plasmid sequences, (ii) plasmids that are merged with or indistinguishable from other replicons, and (iii) plasmids that are erroneously lost [16, 20, 23, 38, 39]. The complete reconstruction of a genome’s plasmids is, however, crucial, as they often encode important adaptive traits, such as antibiotic resistance and virulence [40].

In another study, we successfully resolved three Btt genomes by applying the proposed workflow, revealing a *cry3Aa* gene duplication in two Btt strains [41]. Such genetic differences, even if associated with phenotypic traits, can be missed due to unresolved plasmid sequences, and thus compromise comparative genomics [18].

### False loss of single-copy contigs

In addition to the loss of sequences resulting from algorithmically collapsed repeat regions, genuine single-copy sequences may also be lost in Unicyler’s hybrid assembly. The final genome assembly of Bw EPS29 comprises one circular chromosome (5.4 Mb, GC content = 35.5 %) and two circular plasmids with a size of 557.2 and 277.9 kb, and GC content of 32.6 and 33.0 %, respectively (Table 6). A total of 99.91 % of short reads mapped back to the assembled genome and the BUSCO completeness score was 99.8 % (S: 98.2 %, D: 1.6 %, F: 0.0 %, M: 0.2 %, N: 450).

**Table 6.** Final genome assembly of *B. wiedmannii* strain EPS29

Replicon	Length (bp)	GC (%)	Topology
Chromosome	5 376 035	35.5	Circular
ppl557	557 185	32.6	Circular
ppl278	277 910	33.0	Circular

**Fig. 7.**
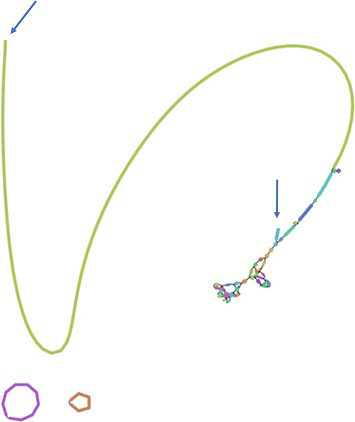
Bandage visualization of the final assembly graph after hybrid auto-assembly with Unicycler. Contigs are represented in boxes and contig connections are visualized by black lines. The auto-assembly of *B. wiedmannii* strain EPS29 contained two complete (circularized) plasmids, but the chromosome was not resolved and consisted of several contigs. Two dead-ends in the assembly graph (marked with blue arrows) indicate missing sequences.

The automatic genome assembly of Bw strain EPS29 resulted in two complete (circularized) plasmids, but the chromosome was not resolved and consisted of several linear contigs (Table 7). The assembly graph of the hybrid auto-assembly revealed that (i) these contigs were somehow connected and (ii) two contigs had a single dead-end, indicating missing sequences (Fig. 7).

**Table 7.** Output of hybrid auto-assembly of *B. wiedmannii* strain EPS29 Only contigs with a minimum size of 1000 bp are shown. The linear contigs highlighted in grey were blast searched in the best SPAdes graph to check multiplicities.

Contig no.	Length (bp)	Topology
1	4 950 916	Linear
2	557 185	Circular
3	277 910	Circular
4	125 082	Linear
5	96 532	Linear
6	68 068	Linear
7	54 264	Linear
8	1446	Linear

A potential false gene loss in the hybrid auto-assembly was supported by BUSCO analysis: the short-read only assembly had a BUSCO completeness score of 99.8 % (S: 98.2 %, D: 1.6 %, F: 0.0 %, M: 0.2 %, N: 450), whereas the hybrid auto-assembly had a completeness score of 88.2 % (S: 86.9 %, D: 1.3 %, F: 0.0 %, M: 11.8 %, N: 450). Thus, 52 BUSCO genes, which were present in the short-read only assembly, were missing in the hybrid assembly.

Next, we examined which contigs present in the short-read only assembly were missing: a blast analysis in Bandage revealed that the hybrid assembly had lost a single 58.3 kb contig. For unknown reasons, this contig (contig name in best SPAdes: 30) did not have a multiplicity assigned to it (appears grey in the best SPAdes graph). Since the 52 BUSCO genes were located on this particular 58.3 kb contig, they were erroneously lost in the hybrid assembly.

**Fig. 8.**
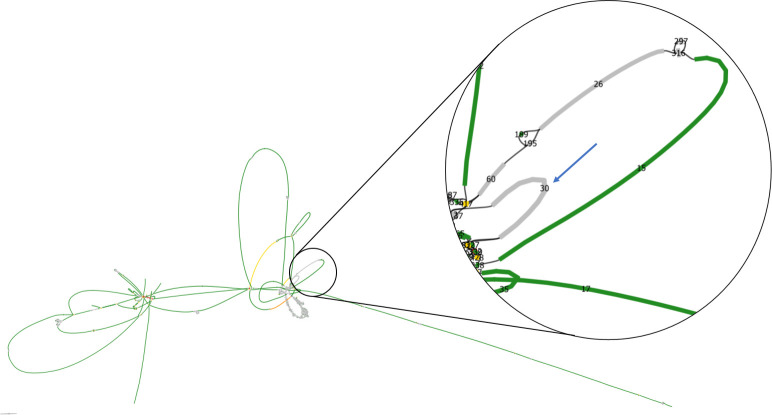
Bandage visualization of the best SPAdes graph of hybrid auto-assembly. Contigs are represented by boxes and contig connections are visualized by black lines. Numbers within boxes indicate contig ID as assigned by Unicycler after short-read assembly. Displaying the graph in custom colours allows us to view Unicylcer’s multiplicity calls (green=single copy, yellow=two copy, orange=three copy, red=four or more copies). Contig 30 (marked with a blue arrow, length=58.3 kb) carries many core genes, but was mistakenly removed by Unicycler in a later step of the pipeline. The best SPAdes graph revealed that contig 30 did not have a multiplicity assigned to it (appears grey).

To prevent Unicycler from removing this contig, we manually set its multiplicity to one (ML:i:1) (see Methods). In close proximity of this contig, two large contigs (contig name in best SPAdes graph: 26; 60) also appeared grey in the best SPAdes graph (Fig. 8). A multiplicity of 1 (ML:i:1) was manually assigned to each of the two contigs (S 26, S 60).

In addition, large linear contigs of the hybrid auto-assembly (contig nos. 4–8, Table 7) were blast searched in the best SPAdes graph to verify the multiplicities of their associated contigs. Two large contigs (S 16, S 32, query contigs 4 and 7, Table 7) were mistakenly classified as repeats. To fix these errors, a multiplicity of 1 was assigned to each of these two contigs (S 16, S 32).

Considering the five corrected multiplicities (Table S3), Unicycler was able to resolve the genome completely (Table 6). The chromosome, which had lost a 58.3 kb sequence during the automatic assembly and was fragmented into several contigs, finally consisted of a single circular contig. The proportion of unmapped reads decreased from 1.6 % (automatic assembly) to 0.09 % (final assembly), and thus 99.91 % of short reads mapped back to the final assembly. Moreover, the final hybrid assembly contained the 52 BUSCO genes that were lost in the automatic approach, resulting in a completeness score of 99.8 %.

In general, BUSCO genes are considered being universally conserved single-copy orthologues, i.e. present in at least 90 % of species within a specific taxonomic group (e.g. *Bacillales*) [31]. Therefore, a large number of missing BUSCO genes is usually a strong indication that something may have gone wrong during assembly and other genes may also be missing. BUSCO quality control after each assembly step (e.g. short-read only assembly, hybrid auto-assembly) helps to identify issues that arise at a specific stage of the pipeline. However, genuine single-copy contigs that do not carry BUSCO genes may also be mistakenly removed in the process of whole genome assembly. If the hybrid assembly graph contains any deadends, it should be verified that genuine contigs of the short-read only (Illumina) assembly are also present in the hybrid assembly. Our analyses illustrate that large single-copy contigs carrying important core genes can be erroneously lost due to unassigned multiplicity. Unicycler is known to remove contaminating or leftover contigs at two stages of its hybrid assembly pipeline [7]: (i) low-depth parts of the short-read only assembly are filtered out to remove contamination; and (ii) leftover sequences are removed during graph cleaning due to insufficient graph-connectivity. Unicycler requires sufficiently long, single-copy contigs, so-called anchor contigs, to scaffold the graph [7]. If large single-copy contigs are not recognized as such, either because they do not have a mulitiplicity or they are wrongly classified as repeats, deffectiveness may be the consequence for the rest of the assembly.

## Conclusion

This study underlines the complexity of bacterial genome assembly, particularly when a hybrid sequencing method is applied. The presented Galaxy-based workflow illustrates both Unicycler’s strengths and limitations in resolving complex bacterial genomes. While the successful genome reconstruction of Btt strain ‘ten BI 256-82/CM3’ and Bw strain EPS29 underlines the importance of integrating short- and long-read sequencing data, our findings highlighted the significant role of manual curation in the assembly process. For Btt strain ‘ten BI 256-82/CM3’, the study revealed the challenges associated with resolving large repeat regions. The analysis demonstrated the algorithmic collapse of an inter-plasmidic repeat, resulting in incomplete plasmid sequences. However, through manual adjustment of contig multiplicities, the workflow successfully resolved the genome. Furthermore, the study identified potential issues with Unicycler’s hybrid assembly, including the risk of losing genuine single-copy contigs. In the case of Bw strain EPS29, it was shown that important single-copy core genes (BUSCO) could be erroneously lost due to unassigned multiplicity. Manual intervention, such as setting correct multiplicities, was necessary to prevent the loss of critical genetic information. The study’s findings contribute to a better understanding of the challenges in genome assembly and highlight the need for quality control of auto-assemblies.

## Supplementary Data

Supplementary material 1
