# Supplementary material for: A practical guide and Galaxy workflow to avoid inter-plasmidic repeat collapse and false gene loss in Unicycler’s hybrid assemblies
Source: Microb Genom. 2024 Jan 10;10(1):001173. doi: 10.1099/mgen.0.001173 (PMC10868617; doi:10.1099/mgen.0.001173)
Supplement: Supplementary material 1 [file mgen-10-1173-s001.pdf]

Supplementary material

1. Supplementary methods

1.1. Visualization of read depth data

Short reads were mapped back to the auto-assembled genome using BWA-MEM. The output file (SAM/BAM) was then used as input for the Galaxy tool Samtools depth (Galaxy Version 1.9) to compute the depth at each position of the genome. Subsequently, the obtained tabular file was used as input for the following R scripts:

```
###Figure 4-------------------------------------------------------------

library(ggplot2)

depth.data <- read.table(file = "PATH/TO/FILE/CM3_false_join.tabular")
colnames(depth.data) <- c("Contig", "POS", "depth")
Con2 <- subset(depth.data, Contig == "2")
#Contig 2 consists of two plasmids which are falsely joined

#Determine sequence length
max.pos <- max(Con2$POS)
min.pos <- 1

#vector for x axis breaks
b <- 20000
s <- seq(0, b*ceiling(max.pos/b), by = b)
s[length(s)] <- max.pos
s[1] <- min.pos
s <- s[-20]

#vector for y axis breaks
bb <- 100
ss <- seq(0, bb*ceiling(max(Con2$depth)/bb), by = bb)

#set colors for the colored rectangles (different sequence regions)
colors <- c("#92D050", "#FFC000", "#0070C0")

#draw the plot for figure 4
p <- ggplot(Con2, aes(x = POS, y= depth)) +
       geom_rect(mapping=aes(xmin=0, xmax = 205000, ymin=-100, ymax=0),
              alpha=0.5, fill = colors[1]) +
       geom_rect(mapping=aes(xmin=205000, xmax = 245000, ymin=-100, ymax=0),
              alpha=0.5, fill = colors[2]) +
       geom_rect(mapping=aes(xmin=245000, xmax = max(s), ymin=-100, ymax=0),
              alpha=0.5, fill = colors[3]) +
       geom_line(linewidth = 0.1)+
       scale_x_continuous(expand = c(0,0), breaks = s) +
       scale_y_continuous(expand = c(0,0), breaks = ss,
                          limits = c(-100, 1200))+
       theme_bw()+
       theme(panel.grid.major = element_blank(),
              panel.grid.minor = element_blank(),
              panel.background = element_blank(),
              axis.line = element_line(colour = "black"),
              strip.background = element_blank(),
              strip.text.x = element_blank(),
              axis.text.x = element_text(angle = 45, hjust = 1))+
       xlab("Contig position [bp]") +
       ylab("Read depth")

print(p)
```

```
###Figure S1-----------------------------------------------------------

library(ggplot2)

depth.data <- read.table(file = "input/FigS1/CM3_linear.tabular")
colnames(depth.data) <- c("Contig", "POS", "depth")
Con2 <- subset(depth.data, Contig == "3")
#Contig 2 consists of two plasmids which are falsely joined

#Determine sequence length
max.pos <- max(Con2$POS)
min.pos <- 1

#vector for x axis breaks
b <- 20000
s <- seq(0, b*ceiling(max.pos/b), by = b)
s[length(s)] <- max.pos
s[1] <- min.pos
s <- s[-20]

#vector for y axis breaks
bb <- 100
ss <- seq(0, bb*ceiling(max(Con2$depth)/bb), by = bb)

#draw the plot for figure S1 (supplementary)
p <- ggplot(Con2, aes(x = POS, y= depth)) +
       geom_line(size = 0.1)+
       scale_x_continuous(expand = c(0,0), breaks = s) +
       scale_y_continuous(expand = c(0,0), breaks = ss, limits = c(-100, 1200)) +
       theme_bw()+
       theme(panel.grid.major = element_blank(),
              panel.grid.minor = element_blank(),
              panel.background = element_blank(),
              axis.line = element_line(colour = "black"),
              strip.background = element_blank(),
              strip.text.x = element_blank(),
              axis.text.x = element_text(angle = 45, hjust = 1))+
       xlab("Contig position [bp]") +
       ylab("Read depth")

print(p)
```

Table S1: Bacterial strains used to count the occurrences of assembly errors. Sequencing data (Illumina + Nanopore) were downloaded from NCBI SRA database and subsequently assembled using the hybrid assembler Unicycler.

**Species**	**Strain or isolate name**	**NCBI BioSample**	**Repeat collapse in Unicycler's auto-assembly**
Klebsiella pneumoniae	KSB1_9D	SAMN07211282	yes
Klebsiella pneumoniae	KSB1_9A	SAMN07211281	yes
Enterobacter hormaechei	C15117	SAMN10174734	yes
Enterobacter hormaechei	MRSN791417	SAMN36773911	yes
Citrobacter freundii	32_P_CF	SAMEA5578852	yes
Citrobacter freundii	30_P_CF	SAMEA5578849	yes
Citrobacter portucalensis	38_P_CF	SAMEA5578862	no
Bacillus thuringiensis ssp. *tenebrionis*	NB125 (DSM 5526)	SAMN31131915	no
Bacillus thuringiensis ssp. *tenebrionis*	BI 256-82 (DSM 2803)	SAMN37105265	yes
Bacillus thuringiensis ssp. *tenebrionis*	ten BI 256-82	SAMN37105266	yes
Bacillus thuringiensis ssp. *tenebrionis*	NB176-1 (DSM 5480)	SAMN31131916	yes
Bacillus thuringiensis ssp. *tenebrionis*	NB176	SAMN31131917	yes
Bacillus thuringiensis ssp. tenebrionis (Novodor)	NB176	SAMN21214336	yes
Bacillus thuringiensis israelensis (Solbac)	BMP144	SAMN21214335	no
*Bacillus thuringiensis ssp. aizawai (*Certan)	B401	SAMN21214334	no
*Bacillus thuringiensis ssp. aizawai (*Agree)	GC-91	SAMN21214333	yes
Bacillus thuringiensis ssp. aizawai (XenTari)	ABTS-1857	SAMN21214332	no
Bacillus thuringiensis ssp. kurstaki (Dipel)	ABTS-351	SAMN21214338	yes

2. Supplementary results

Table S2: Corrected contig multiplicities after hybrid auto-assembly B (ONT read length ≥1,000 bp) of *B. thuringiensis* ssp. *tenebrionis* strain ten BI 256-82/CM3. Contig ID as assigned by Unicycler after short-read assembly is provided. Multiplicities of three contigs belonging to the inter-plasmidic repeat were corrected in the best SPAdes graph. Contig 134 also belongs to the bacterial chromosome, thus a multiplicity of three was manually assigned.

Contig ID	Length [bp]	Multiplicity as assigned by Unicycler	Corrected multiplicity
82	9,839	1	2
94	5,219	1	2
134	1,630	2	3

Table S3: Corrected contig multiplicities after hybrid auto-assembly of *B. wiedmannii* strain EPS29. Contig ID as assigned by Unicycler after short-read assembly is provided. Multiplicities of five contigs were corrected.

Contig ID	Length [bp]	Multiplicity as assigned by Unicycler	Corrected multiplicity
16	125,217	2	1
26	68,588	not assigned	1
30	58,426	not assigned	1
32	54,387	3	1
60	11,729	not assigned	1

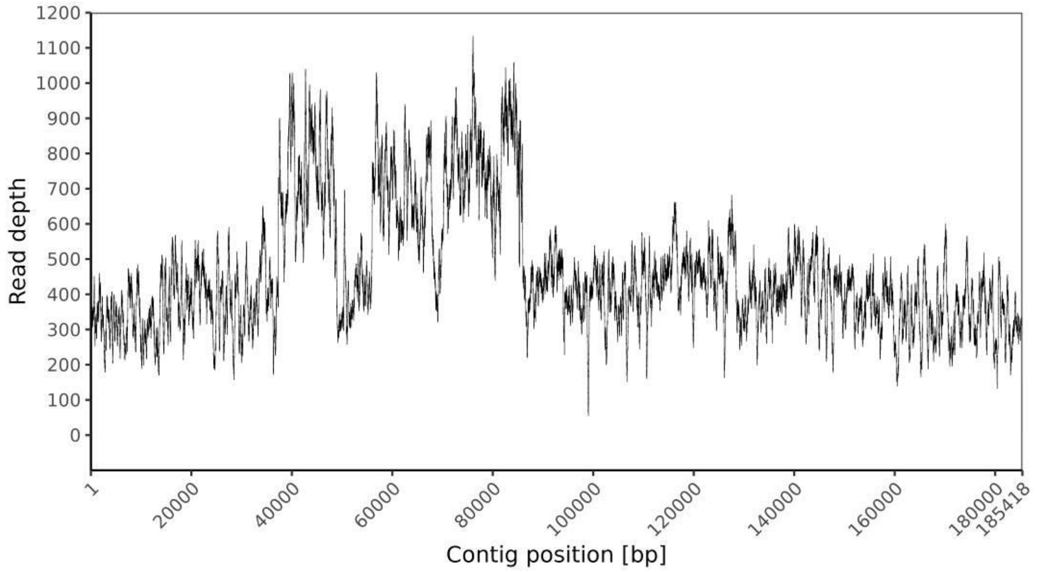

Figure S1: An excessive read depth indicates a repeat region within the circular contig 3B (Table 5). Short reads of Btt strain ten BI 256-82/CM3 were re-mapped against the respective auto-assembly B.
